# A Biophysical Model of Endocannabinoid-Mediated Short Term Depression in Hippocampal Inhibition

**DOI:** 10.1371/journal.pone.0058926

**Published:** 2013-03-18

**Authors:** Margarita Zachariou, Stephen P. H. Alexander, Stephen Coombes, Chris Christodoulou

**Affiliations:** 1 Department of Computer Science, University of Cyprus, Nicosia, Cyprus; 2 School of Biomedical Sciences, University of Nottingham, Nottingham, United Kingdom; 3 School of Mathematical Sciences, University of Nottingham, Nottingham, United Kingdom; University of Sheffield, United Kingdom

## Abstract

Memories are believed to be represented in the synaptic pathways of vastly interconnected networks of neurons. The plasticity of synapses, that is, their strengthening and weakening depending on neuronal activity, is believed to be the basis of learning and establishing memories. An increasing number of studies indicate that endocannabinoids have a widespread action on brain function through modulation of synap–tic transmission and plasticity. Recent experimental studies have characterised the role of endocannabinoids in mediating both short- and long-term synaptic plasticity in various brain regions including the hippocampus, a brain region strongly associated with cognitive functions, such as learning and memory. Here, we present a biophysically plausible model of cannabinoid retrograde signalling at the synaptic level and investigate how this signalling mediates depolarisation induced suppression of inhibition (DSI), a prominent form of short-term synaptic depression in inhibitory transmission in hippocampus. The model successfully captures many of the key characteristics of DSI in the hippocampus, as observed experimentally, with a minimal yet sufficient mathematical description of the major signalling molecules and cascades involved. More specifically, this model serves as a framework to test hypotheses on the factors determining the variability of DSI and investigate under which conditions it can be evoked. The model reveals the frequency and duration bands in which the post-synaptic cell can be sufficiently stimulated to elicit DSI. Moreover, the model provides key insights on how the state of the inhibitory cell modulates DSI according to its firing rate and relative timing to the post-synaptic activation. Thus, it provides concrete suggestions to further investigate experimentally how DSI modulates and is modulated by neuronal activity in the brain. Importantly, this model serves as a stepping stone for future deciphering of the role of endocannabinoids in synaptic transmission as a feedback mechanism both at synaptic and network level.

## Introduction

In the course of millions of years of evolution through natural selection, the human brain has adapted and specialised to perform sophisticated and complex mental functions to ensure our survival. An integral part of these functions is the ability to both learn and adjust our behaviour in response to changes in the environment. Learning can be defined as the ability to acquire and process information from the environment and memory as the ability to retain and recall this information. The changes in the brain that are associated with learning and memory are believed to take place at the level of synapses, the connections between neurons [1]. This capacity of the brain to change with learning is broadly defined as plasticity [2], [3].

More specifically, synaptic plasticity is the ability of a synapse to either become stronger (known as potentiation or facilitation) or weaker (known as depression) as a function of neuronal activity [2], [3]. Plasticity can be classified as short- or long-term, depending on its duration. Short-term potentiation (STP) and depression (STD) can last up to a few seconds, whereas long-term potentiation (LTP) and depression (LTD) have a longer time-scale, from minutes to hours [4]. Both inhibitory and excitatory synapses in the brain are plastic and can undergo short- and long-term changes in their strength [5]. Although the plasticity of inhibitory connections has been mostly overlooked, it has been recently brought into the spotlight (for a comprehensive review see [6]). New evidence is delineating its critical importance in functions such as pain, addiction, memory and learning [6]. Numerous biochemical mechanisms are implicated in synaptic plasticity (excitatory and inhibitory) and can be broadly categorised as changes that occur post-synaptically and/or pre-synaptically [4]. The cannabinoid signalling system has recently emerged as an important modulator of plasticity for both inhibitory and excitatory synapses [7], [8]. Retrograde cannabinoid signalling mediates both short- and long-term forms of homo- and hetero-synaptic plasticity in various brain regions [9].

Cannabinoids (CBs) are broadly defined as a group of chemical substances that activate CB receptors. The three general types of CBs are (a) phyto-CBs, occurring uniquely in the cannabis plant, (b) synthetic CB compounds, produced in the laboratory, and (c) endocannabinoids (eCBs) or endogenous CBs, which naturally form in the body. eCBs underlie an unconventional retrograde signalling system important for many physiological processes , such as pain, appetite and sensory integration [10]. They represent a class of *retrograde* messengers in the sense that they are released post-synaptically and bind to pre-synaptic CB receptors. Currently, there are two known subtypes of CB receptors, CB_1_ and CB_2_ [11], [12]. CB_1_ is the most abundant CB receptor in the brain and can be found in many different areas such as the hippocampus, the neocortex and the amygdala, the basal ganglia and the hypothalamus [10].

One function of eCBs is to regulate neurotransmitter release via activation of pre-synaptic CB_1_ receptors. The activation of CB_1_ receptors reduces the release of the inhibitory neurotransmitter 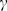-aminobutyric acid (GABA) from interneurons and the release of excitatory neurotransmitter glutamate (GLU) from principal neurons. This phenomenon is known as eCB-dependent short-term depression (eCB-STD) of inhibition (eCB-iSTD) or excitation (eCB-eSTD). Various forms of eCB-STD have been reported which are classified based on the synthesis mode of eCBs [8]. eCB synthesis is stimulated when intracellular levels of Ca^2+^ rise inside the neuron due to cell depolarisation and/or when certain G protein-coupled receptors (GPCRs) are activated. One form of eCB-STD, known as depolarisation-induced suppression of inhibition (DSI), results in reduction of GABA release by retrograde signalling from a strongly depolarised post-synaptic cell to the pre-synaptic GABA-releasing cell [12], [13]. DSI, a widely observed CB-mediated phenomenon, was first reported in hippocampus [14] as well as cerebellum [15]. eCBs can also mediate depolarisation-induced suppression of excitation (DSE) in the hippocampus [12], [13], [16] by suppressing excitatory neurotransmitter GLU release from GLU-releasing cells. However, DSE is suggested to be much less prominent (30 fold) in hippocampus and requires longer depolarisations for induction than DSI [16], supposedly due to the lower expression and sensitivity of CB_1_ receptors on pyramidal cells. Hippocampal DSI and DSE are blocked by CB_1_ antagonists and can be mimicked by application of the CB_1_ receptor agonists [17], [18].

In addition to short-term neuronal plasticity mediated by eCB mobilisation (synthesis and release) following depolarisation (DSE/DSI), a similar inhibition can be induced by activation of certain G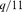-linked receptors. This process is known as metabotropic-induced suppression of excitation (MSE) or inhibition (MSI). The receptors most closely linked to this form of plasticity in hippocampus are the Group I metabotropic glutamate receptors (mGluR), in particular mGlu_1_ and mGlu_5_, and the metabotropic muscarinic receptors (mAChRs), in particular M1 and M3 [19]. Interestingly, MSE/MSI do not seem to require a rise in intracellular Ca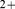 (although it is not completely Ca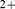 independent as intracellular Ca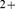 may increase secondary to Inositol Trisphosphate (IP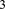) released by Phospholipase C (PLC) during eCB synthesis) [20], [21].

Additional studies suggest that eCB release is involved in the induction of longer lasting forms of plasticity [22]. eCB-iSTD may promote the strengthening of excitatory synapses (LTP) by blocking inhibition of principal cells [12]. The phenomenon of CB-mediated LTD has also been reported: LTD of excitatory transmission in the striatum [23] and LTD of inhibitory synaptic transmission (I-LTD) in the hippocampus [24]. Thus, activation of CB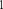 receptors due to repetitive stimulation on glutamatergic axons leads to homo-synaptic LTD on glutamatergic (excitatory) synapses and to hetero-synaptic I-LTD on nearby GABAergic (inhibitory) synapses.

A large body of evidence indicates the importance of the eCB system in the regulation and operation of learning and memory by mediating short- and long-term synaptic efficacy changes [22]. However, the administration of exogenous CBs has been shown to impair learning and memory tasks in both human and animal studies and to inhibit LTP and LTD in hippocampus [25]. Learning and memory impairments have also been reported as a side effect of smoking marijuana, thereby self-administering the active components of CBs [26]. Moreover, exogenous CBs reduce the power of oscillatory brain activity [27], [28], thought to reflect the organisation and coordination of cortical activity amongst different regions of the brain while different types of memory are processed.

Although the pace of research in investigating CB signalling has been accelerating over the past few years, to the best of our knowledge, there are relatively very few theoretical approaches in this particular scientific area [29]–[33]. Currently, all the existing models of eCB dynamics are phenomenological and focus on investigating the CBs role at the network, rather than at the synaptic level. Moreover, to the best of our knowledge, none of the existing models describes the suggested key factors controlling the generation and release of eCBs at post-synaptic sites as well as the pre-synaptic signalling by CB receptors during DSI. Although extensive experiments have characterised many aspects of the eCB-mediated synaptic modulation, there are still many open questions regarding the biochemical signalling molecules and cascades of the eCB system [34], [35], which merit further investigation with both experiments and theory. Hence, the shift of focus to the synaptic level of description of the eCB signalling system comes as a natural step before attempting to further elucidate the mechanism of the network role of CBs in memory and learning.

Our fundamental aim was two-fold: *(1)* to move beyond the existing phenomenological models of the CB retrograde signalling mechanism at the synaptic level [29]–[33] and construct a realistic biophysical model based on existing experimental observations, and *(2)* to use the validated model as a framework to explore the main hypothesis that DSI is the result of fine tuning between the pre- and post-synaptic cells' activities. In this paper, we present the development of such a biophysically plausible model of CB retrograde signalling at the synaptic level and investigate how this mediates DSI in hippocampus, a brain region crucial for learning and memory. In the following sections, we first review the neurobiology of the CB-mediated short-term synaptic plasticity with a focus on DSI. We then provide a short summary of the modelling strategy (with more information in the Methods section) followed by the results and discussion of the *in silico* experiments.

### Neurobiology of Cannabinoid Signalling During DSI

#### eCB Synthesis in the Post-synaptic Cell

The two main eCBs found in the brain are anandamide (AEA) and 2-arachidonoylglycerol (2-AG). eCBs are considered to exist as preformed precursors in the membrane where they are enzymatically produced or “made on demand” in response to specific signals, such as increases in intracellular Ca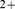 and/or activation of post-synaptic GPCR. However, the notion of “made on demand” eCB synthesis being tightly coupled to its release has been recently challenged and alternative models have been proposed [34], [36]. In general, 2-AG is considered to be more prevalent as a fast retrograde synaptic messenger than AEA. Hence, we focus on the synthesis of 2-AG, which is the main eCB in hippocampus and has been demonstrated to underlie both hippocampal eCB-iSTD and I-LTD [24], [35], [37]–[39].

The main pathway of 2-AG synthesis is considered to involve hydrolysis, by a diacylglycerol lipase (DAGL), of the ester bond at *sn*-1 position of a diacylglycerol (DAG) containing a *sn*-2 arachidonoyl. DAG-driven eCB synthesis can be mediated through two different pathways. One is PLC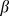-independent and driven by a large increase in intracellular Ca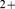 concentration alone, and the other is PLC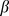-dependent and driven by activation of G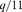-coupled receptor levels [8].

The resultant large increase in intracellular Ca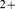 concentration to the micro-molar range (in hippocampus [40]) induces synthesis of DAG through an undetermined pathway that is independent of PLC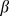 [8], [41], [42]. In the case of eCB synthesis as a result of large Ca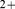 elevations in the post-synaptic cell, the main source of calcium rise is due to Ca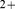 entry through strongly activated voltage-gated calcium channels (VGCCs). Various studies [43]–[51] have shown that the distribution of different types of VGCCs on CA1 pyramidal cells is not uniform. L- and N-type VGCCs are predominantly located on the soma and proximal dendrites whereas T- and R-type VGCCs are predominantly found on the apical dendrites. The rise in intracellular Ca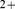 in hippocampal pyramidal cells during DSI is considered to be mainly due to the activity of L-type VGCCs [14]. However, the release of Ca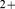 from ryanodine intracellular stores may also contribute to Ca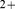-driven eCB release under certain conditions (in particular for younger animals) [52]. Pharmacological activation of NMDA receptors was also found to induce eCB mobilisation and suppression of inhibition [53]. However, it remains to be determined whether local activation of NMDA receptors by synaptically released glutamate is sufficient to induce eCB-iSTD [8]. Moreover, since NMDA receptors are restricted around excitatory post-synaptic sites on the dendrite, the eCB release driven by NMDA receptors may hardly act on perisomatic inhibitory synapses. Thus, it is likely that the NMDA evoked Ca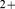-driven eCB mobilisation contributes to synaptic modulation that is spatially limited to a few nearby terminals, whereas the VGCC-mediated mobilisation results in a generalised depression of CB-sensitive incoming GABAergic synaptic terminals [8], [53].

The strong activation of G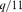-coupled receptors (e.g. mGlu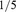) stimulates PLC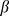, which cleaves Phosphatidyl Inositol Bisphosphate (PIP2) into DAG and Inositol 1,4,5-trisphosphate (IP3). DAG is then converted to 2-AG by DGL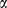 and released to the synaptic cleft. Interestingly, the machinery for synthesising 2-AG through activation of G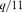-coupled receptors (consisting of mGlu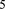, PLC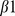, and DGL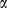) is predominately found at the spines on the dendrites of hippocampal pyramidal cell facing glutamatergic terminals. However, CB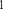 is expressed at very high levels in inhibitory terminals of CCK positive perisomatic basket cells and considerably less in excitatory terminals. Thus, as with the NMDA-mediated eCB mobilisation, it is likely that the mGluR-mediated eCB mobilisation modulates the nearby terminals in a spatially limited fashion. This is consistent with the fact that I-LTD, which is mGluR dependent, is also considered to be limited to GABAergic terminals localised nearby glutamatergic terminals [8], [24].

#### eCB Transport and Degradation

After 2-AG is synthesised, it gains access to the extracellular environment and travels across the synaptic cleft to bind to CB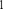 receptors on pre-synaptic cells. It is not clearly understood whether it diffuses through the membranes of the originating cells or is transported across them. 2-AG is then returned into the cells by an as-yet-unknown eCB transporter (by facilitated diffusion or a transporter operating passively [8], [10]), where most of it (85%) is degraded by Monoglyceride Lipase (MGL) [54], [55]. Studies have shown that genetic inhibition of MGL enhances STD of synapses [56], [57]. In the hippocampus, MGL is present pre-synaptically [58] in axon terminals of granule cells and CA3 pyramidal cells throughout their entire axonal arborisation. A subpopulation of inhibitory axon terminals, including those of CCK positive basket cells and axo-axonic cells, also expresses MGL [58]. The most prominent route to degrading 2-AG, other than hydrolysis by MGL, appears to be through oxidative metabolism by enzymes expressed post-synaptically, such as cyclooxygenase type 2 (COX-2) [37], [59]. However, it has been suggested that COX-2 acts within the pyramidal cells by regulating the actual production of 2-AG rather than serving as an elimination step for released 2-AG [60].

#### eCB Uptake and Pre-synaptic Signalling

The CCK positive interneurons, the GABAergic cell population responsible for perisomatic and dendritic inhibition in hippocampus, have distinctive properties including an exclusive dependence on the conotoxin-sensitive N-type VGCCs for release of GABA [60]–[62]. Activation of pre-synaptic CB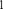 receptors suppresses neurotransmitter release mainly by blocking VGCCs and reducing Ca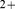 influx into nerve terminals [17], [63]–[65], although K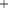 channel activation [66] and direct interference with the release processes can also contribute. The pre-synaptic eCB-iSTD pathway involves a classic membrane-delimited (no cytoplasmic messenger) and voltage-dependent inhibition of pre-synaptic N-type VGCCs, signalled via the G protein 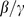 subunit [67].

### Modelling Strategy

**Figure 1:**
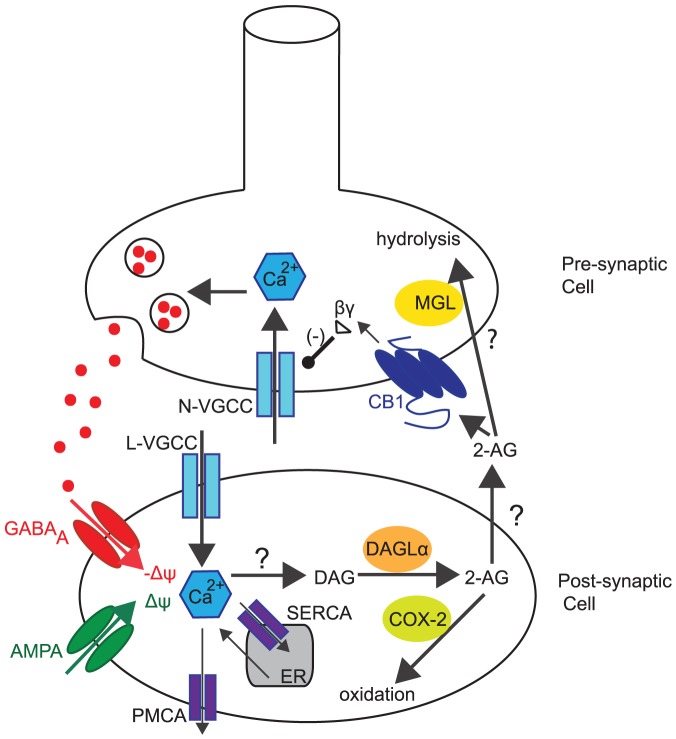
Reduced eCB signalling model at central synapses underlying eCB-iSTD. Highlighting the main factors controlling the physiology of 2-AG at post-synaptic sites of generation and release as well as pre-synaptic signalling by CB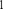 receptors in hippocampus. 2-AG is synthesised in the post-synaptic (excitatory) cell when DAG is metabolised by DGL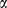. One of the pathways that DAG can be produced is linked to the rise of intracellular calcium concentration, which leads to the production of DAG through an as-yet-unknown pathway. The main source of intracellular calcium post-synaptically is considered to be due to depolarisation (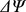) induced entry through L-type VGCCs. A minor source of Ca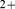 is due to the leak flux from the ER. Ca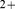 exits the cytoplasm through PMCA and SERCA pumps. Once 2-AG is synthesised, it is released by a putative eCB membrane transporter and activates CB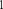 receptors, found pre-synaptically (on the inhibitory cell). This results in the splitting of the G protein heterotrimer into G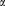 and G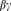 subunits. The G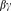 subunit directly inhibits the N-type VGCCs on the pre-synaptic membrane, which puts them into a reluctant state, resulting in the inhibition of GABA neurotransmitter release. 2-AG is then returned into the cells by an as-yet-unknown eCB transporter where most of it is degraded by MGL. Post-synaptically, 2-AG undergoes oxidation by COX-2. Additional information is included in the text. 2-AG: 2-arachidonoylglycerol, COX-2: Cyclooxygenase-2, DAG: Diacylglycerol, DGL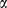: Diacylglycerol Lipase 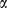, 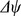: Membrane Depolarisation, ER: Endoplasmic Reticulum, MGL: Monoglyceride Lipase, PLC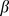: Phospholipase C 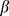, PMCA: plasma membrane Ca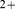 ATP-dependent pumps, VGCC: Voltage-Gated Calcium Channel, SERCA: sarco/endoplasmic reticulum Ca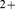 ATP-dependent pumps.

In this paper, we focused on developing a reduced, yet, rich biophysical eCB signalling model underlying DSI at hippocampal inhibitory synapses on excitatory cells. The main components of the model are *(1)* a post-synaptic single compartment model for the excitatory cell with eCB synthesis mediated by intracellular calcium dynamics, and, *(2)* a pre-synaptic single compartment model for the inhibitory cell with CB receptor dynamics modulating inhibitory synaptic transmission. The eCB-iSTD related signalling cascades during DSI and/or agonist administration that are included in the model are illustrated in Figure 1.

Since most experimental studies refer to the somatic DSI (stimulating and recording from the soma), we chose a single compartment model for the post-synaptic cell [31], [68], [69]. We described the intracellular calcium dynamics by adapting a model by Politi et al. [70], in order to include the dynamics for the enzyme DAG (which underlies the eCB 2-AG synthesis) and, also, the L-type VGCC fluxes. We also included kinetic schemes to describe the 2-AG synthesis pathways mediated by calcium rise (with primary source the influx through L-type VGCCs [14]) in the post-synaptic cell. Although it has recently been confirmed that DGL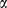 is necessary for DSI [71] for the essential conversion from DAG to 2-AG, the actual pathway which links the post-synaptic Ca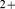 accumulation to the synthesis of DAG remains poorly understood [72]. In our model, we accounted for this pathway by coupling the intracellular calcium concentration to the production of DAG through a Hill's function with Hill coefficient equal to 2 (see equation (37) in the Methods section).

We also modelled the pre-synaptic inhibitory cell with a single compartment [40]. We described the effects of CB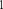 receptor activation in modulating inhibitory transmission by adapting a minimal model for G protein-mediated synaptic facilitation and depression of GABA release via N-type VGCCs modulation by Bertram et al. [73]. The Bertram model is minimal, as it does not include detailed dynamics of G protein activation, the neurotransmitter release process or the pre-synaptic calcium accumulation and calcium channel various states. Nevertheless, it is consistent with previous more detailed models [73]–[75]. By assuming that GABA release is evoked by calcium entry through N-type VGCCs, the simplified model captures the most important features of G protein action, namely *(a)* the G protein-mediated inhibition of N-type calcium channels, and *(b)* the facilitation of the synaptic response of action potential-evoked calcium currents due to a depolarisation-dependent relief of G protein-mediated inhibition of calcium channels. Hence, pre-synaptic activity in our adaptation of the Bertram model [73] results in up-regulation of GABA release (through the dependency of the pre-synaptic VGCCs on membrane depolarisation). Respectively CB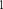 receptor activation results in down-regulation of GABA release (through the dependency of the pre-synaptic VGCCs on the level of the activation of G proteins). It should be noted that the differential activation of the CB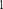 receptor by the eCB (2-AG) and the CB agonist (WIN55,212) was also included in the model, based on experimentally obtained concentration response curves for the CB inhibition of the pre-synaptic VGCCs [76].

In summary, our modelling strategy approach for investigating the CB-mediated modulation of short term inhibitory transmission is minimal, yet, carries significant advantages. Firstly, it highlights the most important mechanisms for eCB-iSTD signalling and, secondly, it offers a reduced model with a small number of equations that can be efficiently incorporated in future network studies.

The main objectives of the present work were to explore the following: *(i)* Can we build a model detailed enough to describe the key experimental characteristics of DSI, including the most essential pathways involved, yet simple enough to use in network studies? *(ii)* Can this model provide suggestions on the variability of the observed DSI, and, *(iii)* Can we use this model to understand the underlying mechanism of the pre- and post-synaptic activity in modulating DSI and provide predictions on the role of the relative timing and magnitude of the cells activities? The above objectives were successfully addressed and the results are presented in the following section.

## Results

The eCB-iSTD phenomenon has been extensively studied experimentally in hippocampal cultures [18], [77] and slices [14], [17] (for an extensive review see [8]). It can be evoked in experiments either by certain modes of electrical stimulation that lead to eCB mobilisation or with the administration of CB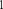 receptor agonist. Here, we focus mainly on experiments done in cultures [16], [18], [78] (although certain results from slices are also considered [40], [67]) from the CA1 region of the hippocampus.

The results presented in this paper are separated in two main stages. In the first phase, we calibrated the model for CB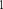 receptor activation to both externally administered exogenous and endogenous CBs. We then investigated whether the model is sufficiently complex to reproduce known characteristics of DSI, a form of eCB-iSTD in hippocampus, such as its short-lived time course, the observed levels of magnitude and its dependency on factors such as the strength of the stimulation and resulting magnitude of intracellular calcium rise. In the second phase, after calibrating the model, we used it as a framework to investigate DSI further under different experimental conditions. The dynamic features of the model were manipulated in order to both clarify the variability across existing experimental data and predict the effect of different experimental paradigms. More specifically, we investigated the following issues: *(1)* the dependency of DSI on calcium dynamics and certain factors by which calcium concentration changes are manifested in the cell, *(2)* the conditions under which DSI can be evoked with physiologically relevant activity, and *(3)* the determining role of the pre-synaptic cell's activity (both magnitude and timing) in modulating DSI.

### eCB-iSTD Magnitude Depends on CB Agonist Concentration

**Figure 2:**
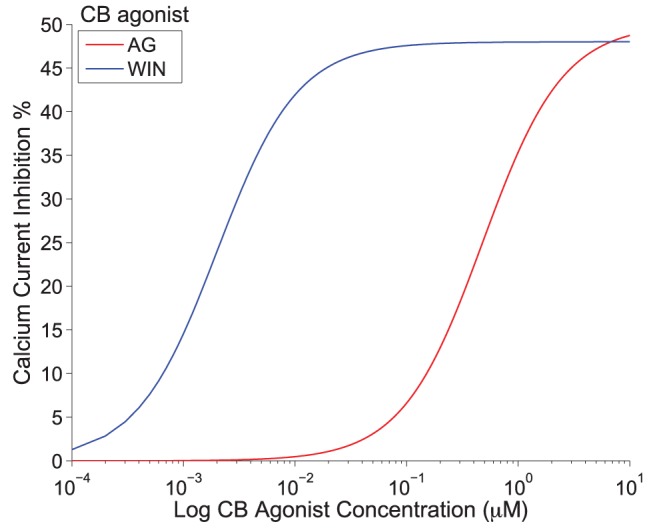
The experimental CB concentration response curves. Hill functions from [76], which correspond to the concentration response curves for the effect of exogenous CB agonist, WIN55,212 (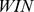), indicated in blue and of the endogenous CB agonist, 2-AG (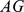), indicated in red, on the pre-synaptic N-type VGCCs on inhibitory CCK positive basket cells.

The CB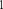 receptor can be activated experimentally either by stimulating the signalling cascades that lead to eCB-mobilisation or by administrating either endogenous (i.e. 2-AG, AEA) or exogenous agonists for the CB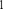 receptor. The most frequently administered exogenous CB agonist for the CB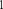 receptor is the WIN55,212 [79]. In order to assess eCB-iSTD during WIN55,212 agonist administration in the model, we used the stimulation Protocol 1 (details in the Methods section). It should be noted that the CB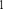 receptor has different efficacies for endogenous and exogenous CBs. In our model, we accounted for this differentiation by including the experimental CB concentration response curves on inhibiting pre-synaptic N-type VGCCs for both 2-AG and WIN55,212 as obtained experimentally in [76]. The Hill functions, which were fitted to these concentration response curves (see equations (15) and (16) in the Methods section), are plotted in Figure 2 as a function of the respective CB agonist concentration.

**Figure 3:**
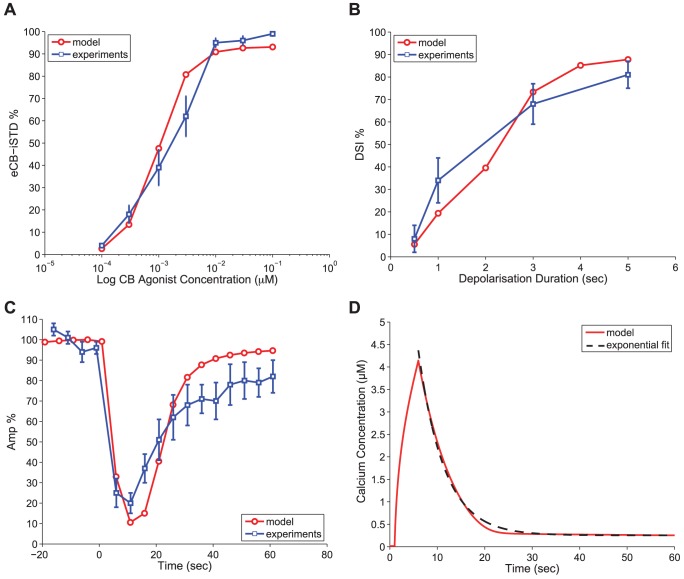
Model validation of eCB-iSTD/DSI characteristics. *Panel A:* eCB-iSTD measure in response to the administration of CB agonist WIN55,212 (Protocol 1) with simulation results in red and experimental results with error bars in blue from [?]. Plotted with a logarithmic scale for the x axis. *Panel B:* The DSI measure in response to post-synaptic depolarisation pulses (0.5–5 sec) (Protocol 2) with simulation results in red and experimental results with error bars in blue from [78]. *Panel C:* The DSI time course from the experiments [18] (in blue) and the model simulations (in red) in response to a 5 sec depolarising pulse (Protocol 2). The amplitude (normalised to the value before depolarisation) of the IPSPs is plotted just before, during and after the depolarising pulse. Decay time constant: 12.81 sec, Peak latency value: 5.92 sec, Peak DSI value: 89.48% (relative amplitude 10.52%). *Panel D:* The calcium concentration time course (in red) from the model simulations in response to a 5 sec depolarising step (Protocol 2). The depolarising step starts at time 1 sec. The exponential fit to the calcium concentration decay from the peak amplitude is superimposed in black (decay time constant 5.43 sec).

The simulation results are shown in Figure 3 - Panel A, in red, in terms of the observed eCB-iSTD due to WIN55,212 administration. eCB-iSTD is measured as the percentage reduction in peak amplitude of the inhibitory post-synaptic potential (IPSP) after the agonist administration (at its minimum value) compared to the average IPSP amplitude before the agonist administration (as detailed in the Methods section). These results are in good agreement with the equivalent experimental data (in blue) from [78] (p. 957 Figure 6 in [78]) for a range of agonist (WIN55,212) concentrations. The maximal eCB-iSTD value, which is defined as the largest asymptotic value obtained for increasing agonist concentration, is about 93%. This is close to the maximal inhibition reported in cultures (as shown in Figure 3 - Panel A) [78] with saturating concentration of WIN55,212 (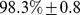) and the maximal value close to 90% reported in slices [17] (from only eCB-sensitive synaptic connections).

**Figure 6:**
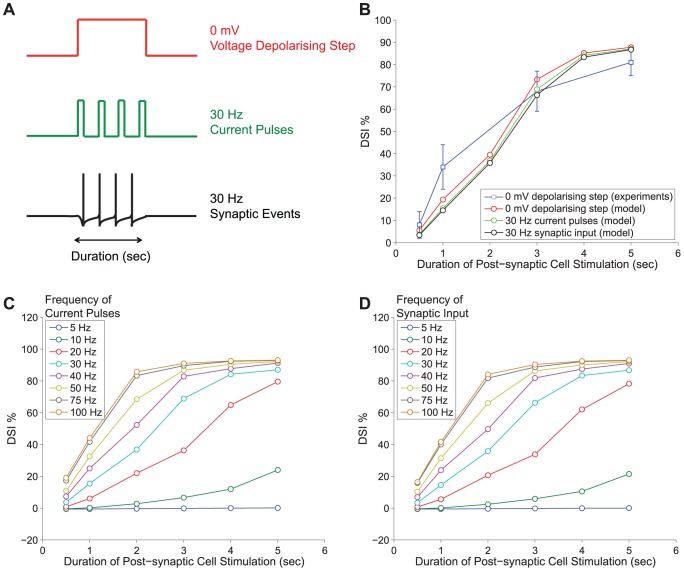
DSI induction with various stimulation protocols. *Panel A:* DSI is induced for various durations of stimulation (0.5–5 sec) with three different protocols; a depolarising voltage step to 0 mV, a 30 Hz train of current pulses (5 msec duration) and a 30 Hz train of action potentials on the excitatory synapse. *Panel B:* The DSI for all three protocols (depolarising voltage step to 0 mV in red, 30 current pulses in green and synaptic input in black) is superimposed on the experimental results from [16], obtained for depolarising voltage steps of varying duration (in blue). *Panel C and D:* DSI is induced for various durations of stimulation (0.5–5 sec) with a train of current pulses, 5 msec duration, (Panel C) and a train of synaptic events (Panel D). The frequency of the stimulatory trains in both cases is varied from 5–100 Hz generating almost identical results (see Protocol 5).

### DSI Magnitude Depends on Depolarisation Duration

DSI is commonly evoked by long-lasting depolarising voltage steps on the post-synaptic cell [8]. Following the calibration of the model in response to exogenous and endogenous CB agonist administration, we shifted our attention to the effect of the duration of the depolarisation step on the magnitude of the evoked DSI. In this section, we juxtaposed the model results in experimental ones from [16] and, hence, we used the same experimental conditions. We ran the simulations with the stimulation Protocol 2 (as detailed in the Methods section), in which the post-synaptic cell's membrane potential is depolarised from -80 mV to 0 mV for various durations (0.5–5 sec) while the pre-synaptic cell is stimulated with positive voltage pulses (80 mV for 2 msec with a 0.2 Hz) to trigger the release of GABA neurotransmitter and generate inhibitory synaptic events. The model results are shown in Figure 3 - Panel B, in red, in terms of the DSI measure (which is similar to the eCB-iSTD measure, see the Methods section). These results are in good agreement with the equivalent experimental data, shown in blue in Figure 3 - Panel B, from [16] (p. 3868, Figure 7C in [16]) for all the different depolarisation durations that were tested. Not only the correct trend was observed of longer depolarisations leading to larger DSI, but also the numerical results were within or close to the error bars given for the average values of the experimental study.

**Figure 7:**
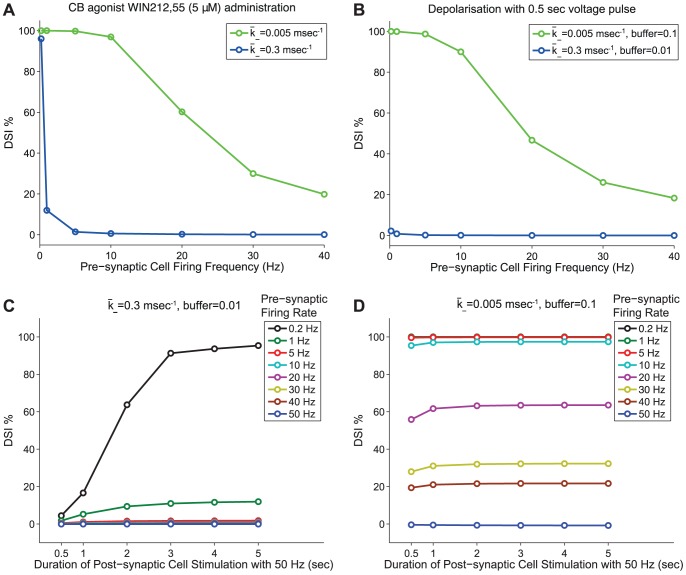
DSI modulation by pre-synaptic firing rate. *Panel A:* DSI as modulated for different pre-synaptic frequencies (0.2–40 Hz) during CB agonist WIN212,55 (5 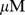) administration (see Protocol 6). Results are plotted in blue for the simulation with 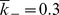 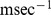 and in green for 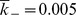 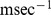. *Panel B:* DSI as modulated for different pre-synaptic frequencies (0.2–40 Hz) while the post-synaptic cell is stimulated a 0.5 sec voltage pulse (see Protocol 6). Results are plotted in blue for the simulation with 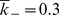 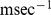, 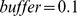 and in green for 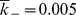 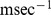, 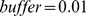. *Panel C:* DSI as modulated for different pre-synaptic frequencies (0.2-50 Hz) while the post-synaptic cell is stimulated with a 50 Hz train for different stimulation durations (0.5-5 sec) of current pulses to elicit DSI (see Protocol 7). Simulations are shown for the set of parameters 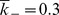 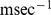, 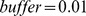. Increasing the pre-synaptic firing induces a strong reduction effect on DSI magnitude for all the durations tested (0.5-5 sec) of post-synaptic stimulations. DSI is completely abolished for pre-synaptic frequencies higher than 5 Hz. *Panel D:* DSI as modulated for different pre-synaptic frequencies (0.2-50 Hz) while the post-synaptic cell is stimulated with a 50 Hz train for different stimulation durations (0.5-5 sec) of current pulses to elicit DSI (see Protocol 7). Simulations are shown for the set of parameters 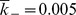 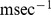, 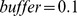. Increasing the pre-synaptic firing induces a strong reduction effect on DSI magnitude for all the durations tested (0.5-5 sec) of post-synaptic stimulations. DSI is completely abolished for high pre-synaptic frequencies such as 50 Hz.

### DSI and Calcium Time Course

DSI is a transient phenomenon that peaks a few seconds after the post-synaptic stimulation and lasts for tens of seconds [80]. The variability of the shape and features of the DSI time course can be seen in various studies from slices and cultures [12], [18], [40], [81]. In order to validate our model dynamics, we compared the model's generated time course of DSI after a 5 sec depolarisation step to the equivalently evoked DSI time course from the experimental study in [18]. The experimental protocol in [18] was the same as in [16], hence, we also implemented the Protocol 2 here (as for the results shown in Figure 3 - Panel B) to obtain the time course of DSI from our model. In Figure 3 - Panel C, we plot in blue the experimental data with error bars from [18] (p. 734, Figure 6B in [18]) and in red the simulated time course of DSI. The DSI time course was obtained by plotting the percentage change of the amplitude (normalised to the value before depolarisation) of the IPSPs just before, during and after the depolarising pulse. The 5 sec depolarising pulse is delivered at time zero. The simulation results follow closely the experimentally obtained trend and many of the main characteristics are captured, such as the peak latency time and the peak DSI value (defined as the time and value at which the greatest percentage reduction of the IPSP amplitude is observed) and the time it takes to return to the baseline (decay time constant).

Various decay time constants have been reported in the literature for the DSI phenomenon. The experimental decay time constant for DSI recorded from cultures at room temperature [18], shown in Figure 3 - Panel C, was fitted with an exponential function to be 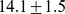 sec. Here, the simulated time course of DSI from our model is 12.81 sec, which falls within the expected time range. It should be noted that in slices (with different experimental protocols) values ranging from 14 to 22 sec were reported for the decay time of DSI (for temperatures of 22-30 °C) [40], [82], [83]. Another interesting feature of DSI is the fact that it decays more slowly than the intracellular calcium in the post-synaptic cell [40], [80]. More specifically, DSI and Ca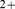 have been found to have decay time constants of 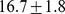 and 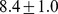 sec in slices [40]. In our model, the calcium decay time constant (5.43 sec) was also considerably less than the DSI one. The calcium time course is shown in red in Figure 3 - Panel D, with the fitted exponential superimposed in black. Although the peak calcium during depolarisation is associated with the DSI magnitude [40], the time course of DSI is not determined by the time course of the calcium transient [84]. The DSI and peak calcium association is further discussed in the following section.

### DSI Depends on Calcium Concentration

**Figure 4:**
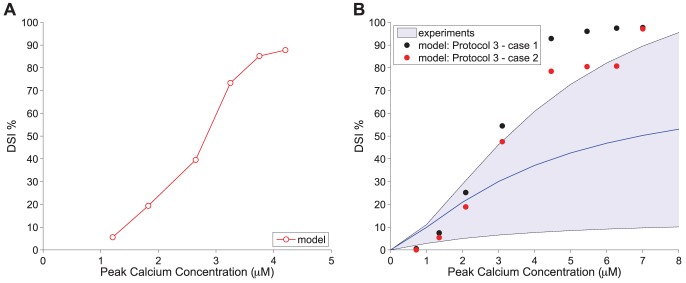
DSI dependence on calcium. *Panel A:* Simulated peak calcium concentrations in response to various depolarisation durations (0.5–5 sec) with Protocol 2, plotted against the respective DSI measure for each depolarisation. *Panel B:* Experimental results from slices [40] showing the fitted Hill function (in blue) of the relation between calcium concentration and DSI. The light blue area indicates the range of maximum and minimum values for the fitted Hill function. The results from the model simulations obtained with Protocol 3 - case 1 are shown in black circles and for Protocol 3 - case 2 in red circles.

The fundamental role of post-synaptic calcium elevation as a necessary and sufficient condition for the synthesis of 2-AG and, hence, the induction of the DSI phenomenon is evident from many studies in both hippocampal cultures and slices [14], [17], [18], [77], [84]. However, the identity of the calcium-sensitive molecule that drives the DAG synthesis and, thus, the 2-AG production during DSI is as yet unknown (for a short review see [72]). In our model, the calcium-DAG pathway is expressed with a Hill's function (see equation (37)) and, due to its sigmoid shape, it shifts from negligible DAG production for basal calcium (0.02 *µ*M) to a substantial one when calcium is increased in the order of several micro-molars. This calcium-DAG relationship is sufficient to reproduce the coupling of calcium elevation to DAG and, hence, 2-AG synthesis in our model. The clear association of DSI to the peak calcium evoked by different depolarisation durations (0.5–5 sec as in Figure 3 - Panel B) can be observed in Figure 4 - Panel A.

In the experimental studies, which we used to calibrate the model from Ohno-Shosaku and co-workers [16], [18], [78] (see Figure 3), the intracellular calcium concentration for different depolarisations was not reported, thus, an equivalent calcium-DSI response curve was not available for comparison purposes with the model in Figure 4 - Panel A. In fact, due to experimental challenges in accurately measuring the concentration of intracellular calcium, calcium measurements are rarely reported in most DSI studies and, in most occasions, when they are reported they are given in terms of a fluorescence imaging ratio and not in terms of concentration. Several factors contributing to the difficulty in quantifying the concentration response curve of DSI as a function of intracellular calcium are discussed in [13]. However, in a study from Wang and Zucker [40], calcium was recorded in terms of concentration ( *µ*M) during a DSI depolarisation paradigm in hippocampal slices. The values of peak calcium reported in the experiments for a 0–5 sec depolarisation were in the range 0–8 *µ*M with a 50% DSI reported to occur at about 3.6–3.9 *µ*M. These experimental results were fitted with a Hill's function 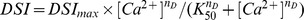 in [40], where 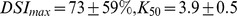 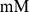 and 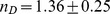 [40].

In order to compare with our model results, we used the stimulation Protocol 3 (see the Methods section), which resembles the experimental conditions used for the Wang and Zucker study [40]. Since the reported values for the calcium concentration are larger (Figure 4 - Panel B) than those we obtained from the model for the same range of depolarisations (Figure 4 - Panel A), we adjusted the buffering parameter 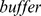 from 0.01 to 0.02 to get similar range of calcium concentrations (for an extensive discussion of the effect of buffering on calcium amplitude and, hence, DSI see the following section on discussing DSI variability due to factors affecting calcium dynamics). It should be noted that the buffering chemical used in the solution for the experiments in [40] was 5 mM 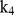
NPE. In Figure 4 - Panel B, we plot the Hill function fitted to the experimental results from [40] (in blue) with the light blue area indicating the range of maximum and minimum values for the fitted Hill function. The simulation results for case 1 (see Protocol 3 in the Methods section) are superimposed in black circles. We then re-plotted the simulation results (case 2 - Protocol 3) for Figure 4 - Panel B with an altered DSI measure. Instead of considering the smallest IPSP following depolarisation relative to the IPSP before depolarisation, we followed the same protocol as Wang and Zucker [40] (see Protocol 3), thus, obtaining lower DSI values (indicated by red circles in Figure 3 - Panel B). We observe that the model results are comparable with the experimental data and within or close to the expected range of values. However, the simulated DSI values for the respective peak calcium values are still in the upper range of values and the adjustments due to Protocol 3 - case 2 (value of depolarisation and DSI measure method) did not change considerably the results. This discrepancy can be attributed to the fact that in the Wang and Zucker study [40] the eCB sensitive synapses were not isolated through paired recording (as in [16], [18], [78]) or pharmacologically. Hence, the DSI measure might be lower than expected due to the fact that only a subset of the recorded synapses is sensitive to eCB and by testing across the entire population results in a smaller DSI magnitude average.

### DSI Variability: Spotlight on Calcium

As discussed in the previous section, the rise of intracellular calcium is an essential and sufficient condition to evoke DSI in hippocampus by triggering the synthesis of 2-AG [14], [17], [18], [40], [77], [84]. Hence, it would be reasonable to assume that post-synaptic factors controlling the calcium dynamics and, in particular, the peak calcium will also heavily determine the concentration of 2-AG and, hence, the observed DSI. In a recent study [85], the differences between calcium-driven plasticity outcomes in various preparations (such as hippocampal cultures and hippocampal and cortical slices) were suggested to arise due to differences in parameters controlling the calcium dynamics. Due to the importance of the peak calcium in DSI, here we focus on factors affecting the peak calcium concentration, such as endogenous and exogenous buffering factors [84] and the availability of extracellular calcium concentration, which can alter the peak amplitude of the intracellular calcium rise. However, other factors that regulate 2-AG synthesis post-synaptically, such as the availability of the enzyme COX-2 and the substrate availability of DAG, could also affect the variability of DSI.

**Figure 5:**
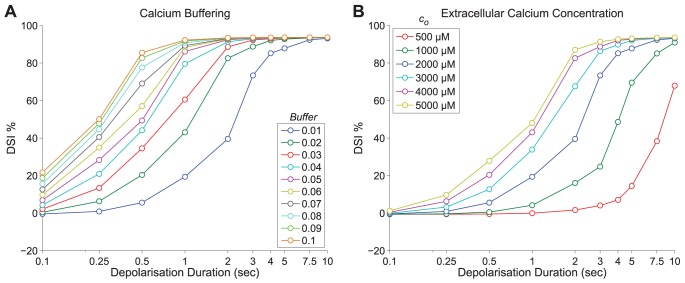
Calcium buffering and extracellular calcium. *Panel A:* The effect of varying the calcium buffering parameter on DSI for various depolarisation intervals 0.1–10 sec (see Protocol 4) plotted with a logarithmic scale for the x axis. Buffering (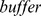) varies from 0.01–0.1 (equivalent to 99–90%) and is colour coded accordingly. *Panel B:* The effect of varying the extracellular calcium (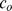) parameter on DSI for various depolarisation intervals 0.1–10 sec (see Protocol 4) is plotted with a logarithmic scale for the x axis. Extracellular calcium varies from 500–5000 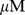 and is colour coded accordingly. It should be noted that in both Panels A and B the blue line represents the standard set of parameters (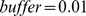, 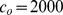 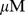).

Here, we implemented Protocol 4 (see the Methods section). Firstly, we varied the buffering coefficient 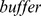 (see equation (39)), which represents the fast intracellular calcium buffering and takes values between 0 and 1, with 1 being equivalent to no buffering and 0 to complete buffering. The custom value for the buffering was taken to be 0.01 (that is, only 1% of the calcium current through VGCCs is actually available to change the concentration of free Ca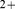 ions in the cell) [86]. Figure 5 - Panel A shows the effect of varying the buffering in the model-generated DSI. We observe that in the presence of less buffering (hence higher values of 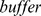) the activation curve shits to the left, that is, DSI can be evoked with shorter depolarisation voltage steps. This is consistent with the results first reported from Lenz and Alger [84] showing that by lowering the calcium buffers, BAPTA or EGTA, used at mM concentrations in experiments on DSI can greatly enhance the ability of short duration voltage steps to elicit DSI. For example, in a study from Ohno-Shosaku et al., [16], with buffering 0.2 mM EGTA, a 0.5 sec depolarising step induced a mere DSI 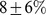 (as seen in Figure 3 - Panel B) equivalent to the results with 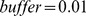 in our model (Figure 5 - Panel A). In contrast, in a study from Földy et al., (in slices) [67], with lower buffering (0.05 mM EGTA), the same depolarisation (0.5 sec) induced DSI close to 100%, which would be better described with less buffering (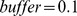) by our model (Figure 5 - Panel A). In the section “DSI Depends on Calcium Concentration”, we also adjusted the buffering parameter 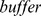 from 0.01 to 0.02 in order to obtain the same range of peak calcium values as observed in the Wang and Zucker study [40]. Effectively, the manipulation of the buffering capacity of the intracellular medium enabled us to describe the peak calcium-DSI response curve across different experiments [16], [40], [67].

The available extracellular calcium can also affect the dynamics of the intracellular calcium concentration. Experiments in hippocampal cultures have shown that the induction of DSI requires the presence of extracellular calcium [77]. In most experiments the external calcium concentration lies in the 2000–2500 *µ*M range. It should be noted that, in all the studies in the previous sections to which we calibrated the model [16], [78], the extracellular calcium concentration was the same (2000 *µ*M). Here, we further tested the DSI responsiveness in the model by varying the extracellular calcium concentration (the results are shown in Figure 5 - Panel B). We observe that when the extracellular calcium is increased to 5000 *µ*M, the evoked DSI magnitude increases due to a certain voltage step (as reported by [82], [84]), whereas the contrary takes place when it is reduced to 500 *µ*M.

### DSI Induction with Physiologically Relevant Neuronal Activity

DSI is commonly induced under non-physiological conditions with long-lasting (in the order of seconds) voltage depolarising steps resulting in an intracellular calcium rise, which in turn triggers the sub-cellular pathways leading to production of 2-AG. However, such stimulation protocols are artificial and not encountered in the brain. Thus, it is important to study how DSI is evoked with more physiologically relevant stimulation in order to assess the *in vivo* role of DSI. In fact, it has been suggested that normal action potential firing patterns of CA1 cells are not sufficient to cause eCB-mediated DSI [87], although in a later study, synchronous and convergent synaptic inputs from multiple sources (with a cumulative frequency of 30 Hz) were shown to be effective in triggering DSI [88].

In both culture and slice experiments, it has been demonstrated that DSI can be evoked with a train of short depolarising current pulses [14], [18], [88]. For example, in a study by Ohno-Shosaku et al. [18], the stimulation of the post-synaptic cell with 50 Hz current pulses (of 5 msec) for 3 and 5 sec yields comparable (yet not the same) DSI as in the case of 5 sec voltage depolarisation to 0 mV. The experimental conditions in [18] were similar to the ones the model was previously calibrated to [16], [78]. Hence, we also tested whether the model yields similar DSI when the post-synaptic cell is stimulated with short current pulses instead of long voltage depolarising steps. In addition, we use a third and more physiologically relevant version of eliciting DSI, that is, by driving the excitatory synapse on the post-synaptic cell by a train of action potentials. It should be noted that in this case the strength of the synapse is such that an action potential per synaptic event will be elicited post-synaptically, which is not realistic as originating from one terminal. Hence, it can be considered as the simultaneous synchronous overlapping input from a population of neurons. The three modes of DSI induction can be visualised in the diagram in Figure 6 - Panel A.

For this set of simulations we held the pre-synaptic frequency to 0.2 Hz as before and we presented varying duration and frequency activation pulses/synaptic events to the post-synaptic cell to evoke a train of action potentials. The stimulation protocol implemented here (see Protocol 5 in the Methods section) follows the experiments in [18] and the current amplitude/peak synaptic conductance was adjusted so that it was sufficient to elicit an action potential on the post-synaptic cell (as in [14]). In addition, we ran the simulations for a range of frequencies (5–100 Hz) and durations (0.5–5 sec) for the stimulation input train on the post-synaptic cell. The collective results can be seen in Figure 6 (with current pulses in Panel C and synaptic input in Panel D).

A first observation is that the current pulses and the synaptic events produce almost identical results and that both methods are able to generate comparable DSI to the depolarisation method. Another observation is that the minimal frequency to obtain DSI was 10 Hz, but only for longer intervals. If we consider significant DSI to be higher than 10%, then for 10 Hz this can be obtained only with intervals longer than 4 sec. Another interesting observation is that for frequencies greater than 75 Hz, DSI saturated in the sense that any frequency above 75 Hz resulted to similar DSI for the respective duration of the stimulus.

Moreover, the simulations in this range of frequencies revealed that the optimal frequency for obtaining the same DSI for the 0.5–5 sec duration of activation for the three modes lies in the 20–50 Hz interval, with 30 Hz being the optimal fit. This can be seen in Figure 6 - Panel A, where we sketch all three modes of activation (depolarisation, current pulses, synaptic events) and the equivalent evoked DSI in each case for different stimulation intervals (Figure 6 - Panel B). The experimental results from Figure 3 - Panel B are superimposed for comparison purposes in Figure 6 - Panel B.

### DSI Depends on the Activity of the Pre-synaptic Cell

The activity (e.g. firing frequency) of the post-synaptic cell reportedly determines the magnitude of DSI [88]. Interestingly, it has been demonstrated that the state of the pre-synaptic cell and, more specifically, its firing rate can also modulate the magnitude of DSI [67]. Földy et al. have suggested that the pre-synaptic activity can overcome CB receptor signalling through the voltage-dependent removal of G protein-mediated inhibition of pre-synaptic calcium channels (a dependency which is retained in our model, as shown in expression (11) in the Methods section). More specifically, experiments have demonstrated that pre-synaptic activity greater than 20 Hz was able to relieve inhibition of GABA release in the case of CB agonist WIN55,212 administration (5 µM), whereas strong DSI was preserved when the pre-synaptic cell was firing with 10 Hz [67]. Our model (as calibrated with [16], [18], [78] and stimulation Protocol 6 - see the Methods section) did not reproduce these results; as seen (in blue) in Figure 7 - Panel A, DSI was completely abolished for all pre-synaptic frequencies greater than 5 Hz. This discrepancy suggests that the dynamics controlling GABA release were more sensitive to CB receptor activation and less sensitive to pre-synaptic firing rate in the Földy et al. experiment [67] than in the Ohno-Shosaku et al. studies [16], [18], [78]. To test this hypothesis, we reduced the parameter 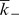 from 0.3 to 0.005 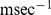 and re-ran the simulation. The obtained results (Figure 7 - Panel A, in green) were consistent with the experiments; strong DSI was still preserved with a pre-synaptic 10 Hz frequency and higher frequencies of more than 20 Hz, were required to strongly reduce DSI.

In another experiment in Földy et al. [67], 500-ms-long depolarizations to 0 mV in the postsynaptic cell evoked strong DSI that was still preserved with a pre-synaptic 10 Hz frequency, whereas a pre-synaptic frequency of 40 Hz was able to strongly reduce DSI. Our model (as calibrated with [16], [18], [78] and stimulation Protocol 6 - see the Methods section) did not reproduce these results, as seen (in blue) in Figure 7 - Panel B, as the depolarisation was too short to induce DSI. This indicates that in the Földy experiments DSI was induced with much shorter depolarisation pulses, as discussed in section “DSI Variability: Spotlight on Calcium”. To accommodate this in our model, we changed the buffering parameter, 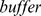, from 0.01 to 0.1. In Figure 7 - Panel B, we plot (in green) the results of the model with the set of modified parameters (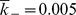 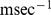, 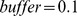) and observe that the model is again consistent with experiments; strong DSI was still preserved with a pre-synaptic 10 Hz frequency, whereas a pre-synaptic frequency of 40 Hz was able to strongly reduce DSI.

Finally, we tested the effect of the constant firing frequency (0.2–100 Hz) of the pre-synaptic cell on the DSI magnitude for both parameter sets; firstly with 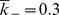 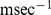, 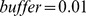 and, secondly, with 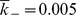 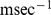, 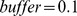. For the fixed post-synaptic stimulation with current pulses, we choose 50 Hz, which provides an example of observable DSI for all duration intervals (indicated in Figure 6 - Panel D). Note that in all the previous simulations for model validation the pre-synaptic firing rate was set to the default low frequency of 0.2 Hz as in the experimental studies [16], [18], [78]. For more information on the stimulation protocol implemented here see Protocol 7 in the Methods section. The modulation of DSI for the 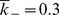 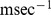, 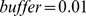 parameter set with respect to the different pre-synaptic frequencies can be seen in Figure 7 - Panel C. Increasing the pre-synaptic firing induced a strong reduction effect on DSI magnitude for all the durations of post-synaptic stimulation that were tested here. In fact, DSI was completely abolished for pre-synaptic frequencies higher than 5 Hz. In contrast, for the 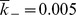 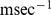, 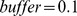 parameter set, DSI required much higher frequencies (e.g. 50 Hz) to be abolished, as seen in Figure 7 - Panel D. Hence, our simulations reveal that not only the CB availability but also the state (i.e. the firing rate) of the inhibitory cell determines whether DSI will be evoked and at which magnitude [67].

### DSI Modulation by the Timing of Pre- and Post-Synaptic Cells' Activity

In the previous subsection, we explored how the state of the pre-synaptic inhibitory cell and in particular its constant firing frequency affects the ability of the synapse to undergo DSI and the effect on the resulting DSI magnitude. However, interneurons (and pyramidal cells) do not normally fire in a periodic sustained fashion *in vivo*. Instead, their firing pattern is irregular characterised by an occasional occurrence of short bursts of high frequency activity. In fact, in [67] a short burst of activity (15 action potentials of 100 Hz) was able to completely reverse DSI when delivered during the period of complete DSI right after the post-synaptic stimulation.

**Figure 8:**
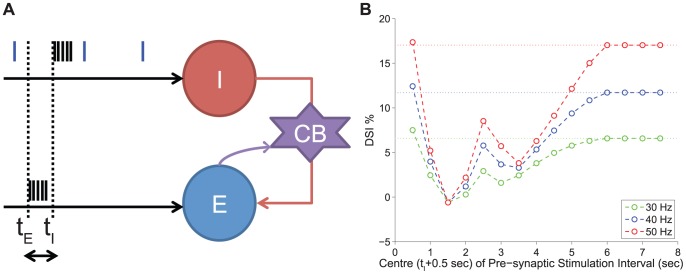
DSI modulation by the timing of pre- and post- synaptic cells' activity. *Panel A:* The illustration of the *in silico* experimental Protocol 8. A signal (1 sec long) arrives at both the inhibitory pre-synaptic cell (indicated by the cell I) and excitatory post-synaptic cell (indicated by cell E) and, thus, evoking a train of action potentials (black spikes). The signal arrives at the two cells with a time difference 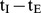. As in experiments with pair recordings, a test current pulse of 0.2 Hz is also applied on the pre-synaptic cell, for assessment of DSI (indicated by the blue spikes). The pre-synaptic cell inhibits the post-synaptic cell whereas the post-synaptic cell under certain conditions mobilises eCBs, activates the CB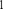 receptor and as a result reduces the inhibition it receives. *Panel B:* The post-synaptic cell receives input always at 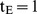 sec and the pre-synaptic cell receives input at different times indicated by 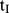. The DSI magnitude is plotted (indicated by the open circles) versus the centre (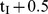 sec) of the time window within which the pre-synaptic cell receives input. The same protocol is applied to the two cells for different frequencies in the range of 30–50 Hz. Depending on the arrival time of the signal at the pre-synaptic cell, the modulation of DSI magnitude differs. When the two bursts coincide they cancel out and, hence, DSI is completely abolished. The maximal effect of DSI, other than this case, occurs with certain latency for the frequencies 30–50 Hz. The horizontal lines indicate the basal value on DSI in each frequency case (i.e. the DSI due to 1 sec firing of the post-synaptic cell with a given frequency, in the absence of input to the pre-synaptic cell). See the text for more information.

In order to investigate how DSI would be modulated for two cells embedded into a network and receiving non-tonic input, we considered the following experimental scenario (for implementation details see Protocol 8 in the Methods section). We assume an incoming (e.g. sensory) signal arrives at both the pre-synaptic and the post-synaptic cells and, thus, evokes a train of action potentials. There are three broad time bands in terms of the relative timing of arrival, that is, *(1)* the signal arrives to both cells at the same time, *(2)* the signal arrives first to the pre- and then to the post-synaptic cell, and *(3)* the signal arrives first to the post- and then to the pre-synaptic cell. In order to investigate the effect of timing of the incoming signal to the two cells, we considered the duration and the magnitude (frequency) of the signal to be the same, representing a common source. The arrival time of the signal to the post-synaptic cell was held constant (at time 1 sec) and the timing of arrival to the pre-synaptic cell was considered for various times before, during and after the signal has arrived to the post-synaptic cell (with the window of activation shifting along the time axis for each trial). We considered a 1 sec long incoming stimulating signal that evokes action potentials in both the pre- and the post-synaptic cells. As in experiments with pair recordings, a test current pulse of 0.2 Hz was also applied on the pre-synaptic cell, for assessment of DSI. An illustration of the experimental scenario can be seen in Figure 8 - Panel A. The two cells receive a set of current pulses evoking spikes to both cells (indicated by the black spikes). The pre-synaptic cell receives an additional test current pulse of 0.2 Hz (indicated by the blue spikes).

The simulation results of this experimental scenario are presented in Figure 8 - Panel B for three example cases with different frequencies of stimulation (30, 40, 50 Hz). The DSI magnitude is plotted versus the centre of the time window within which the pre-synaptic cell receives input (for example, 0.5 sec corresponds to 0–1 sec stimulation etc.). We observe that DSI is decreased in varying degrees, depending on the arrival time of the signal at the pre-synaptic cell with reference to the arrival at the post-synaptic cell. The observed phenomenon was consistent across a range of frequencies of the incoming signal to the two cells (see Figure 8 - Panel B). Horizontal lines indicate the basal value on DSI in each frequency case, that is, the DSI due to 1 sec firing of the post-synaptic cell with a given frequency in the absence of input (other than the test pulse of 0.2 Hz) to the pre-synaptic cell.

**Figure 9:**
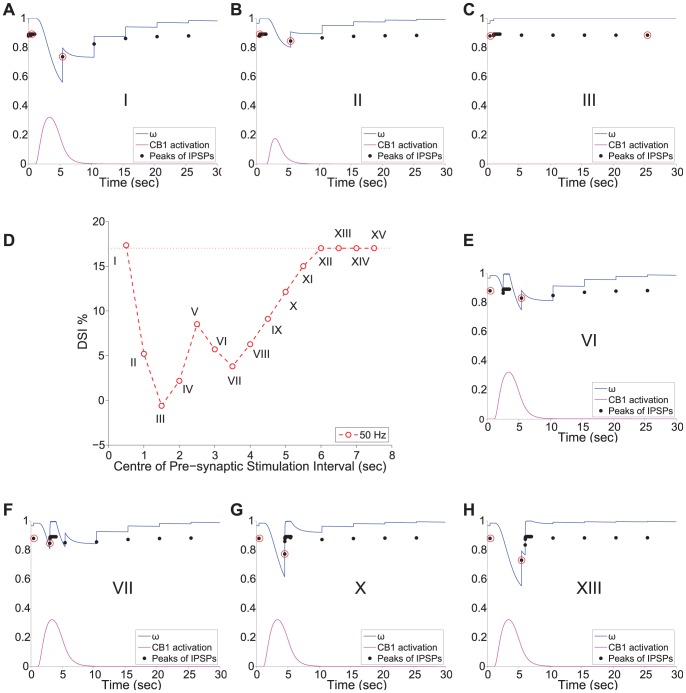
DSI modulation by the timing of pre- and post- synaptic cells' activity - Spotlight on 50 Hz case. *Panel D*: Re-plotting the results from Figure 8 - Panel B for the case in which the 1 sec input to the two cells is 50 Hz. Roman numbers I-XV mark each different sub-case of pre-synaptic input at a different time relative to the input at the post-synaptic cell. The horizontal line indicates the basal value on DSI for the 50 Hz Case. *Panels A-C and E-H*: Plots of the fraction of willing Ca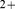 channels (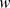) in blue, the CB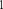 receptor activation (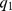) in purple and the amplitude of the IPSPs (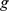) in black dots, for a few of the sub-cases indicated by roman numbers I-XV on Panel D. The red circles indicate the average pre-stimulation IPSP and the minimum post-stimulus IPSP. See the text for more information.

The main observation is that DSI is modulated depending on the relative timing of the pre- and post-synaptic cells' burst of activity. In order to illustrate how exactly the relative timing of the pre- and post-synaptic cells' activity modulates DSI, we re-plot the results for the case when the frequency of stimulation is 50 Hz in Figure 9 - Panel D, along with additional figures showing for several cases of different time-lags the effect on the CB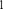 receptor activation (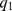 - equation (13)), the fraction of willing Ca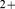 channels (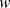 - equation (10)), and the amplitude of peaks of the IPSPs (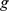 - equation (28)). The maximal depression of DSI at zero-lag (mark III in Figure 9 - Panel D) is explained by the fact that in this case, the activity of the pre- and post-synaptic cells fully coincides in the 1–2 sec interval. As a result of the signals arriving simultaneously, the post-synaptic cell is prevented from firing and inducing DSI in the first place (Figure 9 - Panel C). Interestingly, when the pre-synaptic activation interval overlaps partially with the post-synaptic one (0.5–1–5 and 1.5–2.5 sec, marked by II and IV in Figure 9 - Panel D) the firing of the postsynaptic cell is partly inhibited and, hence, CB receptor activation and, as a result, DSI are lower than in the basal case. This can be observed from the lower CB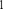 receptor activation (indicated in purple in Panel B).

We also observe that the maximum DSI, which is close or equal to the basal DSI, is recorded in two cases. In the first instance, DSI is at its maximum value when the input arrives first to the pre-synaptic cell and then to the post-synaptic cell (mark I in Figure 9 - Panel D). This is due to the fact that the potentiation of the GABA synapse takes place before the effect of CB begins, when 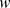 is close to maximum (steady state). Thus, the average amplitude of the IPSP is slightly larger as the pre-synaptic firing induces potentiation and, hence, larger pre-stimulus IPSPs without influencing the CB effect on IPSP post-stimulus, hence the slightly larger DSI (Figure 9 - Panel A). In the second instance, DSI is at its maximum value for pre-synaptic firing in the interval 6-10 sec (Figure 9, marks XII–XV). This is due to the fact that the IPSP with the lower amplitude, which is taken into account for DSI, is recorded at 5.5 sec. Hence, any subsequent input has no impact on the DSI magnitude. This phenomenon is illustrated in an example case in Figure 9 - Panel H.

The shape of the DSI with regards to the relative timing of pre- to post-synaptic cells' activity appears counter-intuitive at first in the 2-6 sec interval of activation. We observe an inverse relation of the time interval that leads to the maximal inhibition of DSI to the activation curve of the CB receptor. This phenomenon can be explained by considering two phases in more detail, with the first phase being the time interval before the CB receptor activation peaks and the second being after the CB receptor activation peaks. In the first phase, the input from the pre-synaptic cell counteracts the effect of the CB receptor activation on 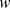, which is shifted to maximum value 1 (due to persistent pre-synaptic firing) and starts decaying at a later point, hence, resulting to a progressively higher amplitude IPSP and a lower DSI measure (as illustrated in Figure 9 - Panel E). In contrast, in the second phase, any interval of pre-synaptic activation from the peak onwards results in one of the IPSPs of the 1 sec stimulus being the minimum IPSP taken into account for DSI as oppose to the second test pulse given at 5.5 sec (as illustrated in Figure 9 - Panels F, G).

These results provide important insights to the conditions that modulate DSI at the synaptic level. In accordance with the results from [67], in which a short burst of action potentials from the pre-synaptic cell can overcome even complete DSI, in our *in silico* experiments a short burst of activity has a similar impact. Most importantly, our analysis clarifies that such an effect is maximal when the pre-synaptic activity coincides with the peak of the CB receptor activation. The particulars of the duration of such a burst will depend on the magnitude of the eCB mobilisation and CB availability among other factors that determine the DSI time course. In summary, our findings reveal the importance of the timing of the pre-synaptic cell burst of activity, with relation to the CB receptor activation and offer an intuitive explanation of each stage.

## Discussion

In the present work, we have shown with *in silico* experiments that the eCB system short-term modulation of synaptic function in brain areas involved in memory and learning (such as the hippocampus) results from the orchestrated action of mechanisms distributed both pre- and post-synaptically. The development of such a model can serve as the main component for exploring the role of CB signalling at the synaptic level on network behaviour associated with learning and memory tasks.

We have formalised the description of the key signalling cascades and molecules that are known to underlie hippocampal DSI, a prominent eCB-iSTD phenomenon. We focused on the main subcellular processes underlying 2-AG mobilisation, uptake and degradation. To the best of our knowledge, this is the first extensive and biophysically rich model of the DSI mechanism that accounts for both the intracellular calcium dynamics and signalling pathways for 2-AG synthesis post-synaptically and, also, for the CB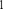 receptor-mediated effect in regulating the VGCC-dependent inhibitory transmission pre-synaptically. We have demonstrated with *in silico* experiments that this model is a mechanistically sound platform that is able to reproduce DSI results from several experimental studies [16], [18], [78] without the use of rigorous optimisation. In particular, the model reproduced several well-known features of the kinetics of DSI in hippocampus, such as its time course and magnitude dynamics. It also highlighted the necessity and sufficiency of intracellular calcium concentration rise in eliciting DSI through 2-AG mobilisation, as shown in various experimental studies [14], [17], [18], [40], [77], [84]. A simple description of the as-yet-unknown calcium-DAG dependency was sufficient to capture the calcium-driven 2-AG synthesis.

The validated model has served not only as a platform for integrating the knowledge about different features of DSI but also for unifying the experimental results from different studies in a consistent manner [16], [18], [40], [67], [78]. During the model formulation process, we were able to reveal hidden contradictions and inconsistencies in the existing experimental studies. Hippocampal DSI has been extensively studied with various experimental preparations both in cultures and slices [8]. However, reported values of DSI features, such as maximal DSI, time course and minimum activation required to evoke it, are not always consistent across studies. For example, the same voltage step can be more efficient in triggering DSI or result in significantly different DSI magnitudes. This observed variability of the DSI phenomenon could be due to differences in experimental protocols [13], [77] and/or due to the expected heterogeneity in neurons pre- and post-synaptically. Experimental factors can include differences in intracellular solutions, animal age, isolation or not of only CB-sensitive synaptic terminals, blocking or not of mGluR-mediated STD (MSI) and choice of the method for evoking DSI (by spontaneous or evoked IPSPs) [13]. In terms of the pre-synaptic cell, factors that could be responsible for these discrepancies include the CB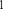 receptor level of expression and sensitivity [10] and the availability and dynamics of the degrading enzyme MGL [39]. Since the magnitude of DSI markedly depends on the post-synaptic peak calcium levels, we used our model as a framework to investigate certain key factors that can alter the amplitude of the peak calcium concentration [84]. Direct numerical simulations of the model suggest that the intracellular buffering and the extracellular calcium concentration are important factors that determine the depolarisation-mediated peak calcium concentration and, hence, the value of DSI. In fact, our findings suggest that calcium buffering is one of the important variability factors that can reproduce experimental data from different preparations (hippocampal cultures and slices) regarding the peak calcium-DSI response curve [16], [40]. In addition, by modifying two key parameters which determine the intracellular calcium buffering and the CB receptor sensitivity to pre-synaptic firing, we were able to reproduce the results from an additional study in slices [67], indicating both the robustness of the model and the variability across experimental data.

Our simulations have also highlighted another discrepancy among experimental protocols, namely the different methodology in assessing DSI. In the Wang and Zucker study [40], DSI was measured by considering the mean of the first two synaptic currents just after the end of the depolarising pulse relative to the synaptic currents before the depolarisation. However, in the experiments by Ohno-Shosaku and co-workers [16], [18], [78], in which higher DSI values were reported, DSI was measured considering the mean amplitude of synaptic currents acquired between 4 and 18 sec (or 6-16 sec) after the end of the depolarising pulse relative to that acquired before the depolarisation. Our simulations suggest that the choice of DSI measure can indeed result in different values, depending on the effective time-course of DSI (it has been reported that DSI takes on average 0.5-1.5 sec to start and several more seconds to peak [80]).

We also used the calibrated CB signalling model as a framework to investigate what is the minimum length and frequency activation required to obtain observable DSI (defined here as higher than a certain percentage, for example 10%). Hence, we firstly established that the model can produce DSI not just with strong and long voltage depolarising pulses (in the order of seconds), but also with short current pulses that can evoke action potentials at the post-synaptic cell or with synaptic activity. Our simulations indicated under which conditions (duration and frequency bands of stimulation) of physiologically relevant post-synaptic activation DSI can be evoked. More specifically, the optimal frequency for obtaining DSI in the same duration range as with the depolarising voltage steps was revealed to lie in the 20-50 Hz interval, with 30 Hz being the optimal fit. This was the case for action potentials evoked with both current pulses and synaptic events. Moreover, our results indicate that for high enough frequencies (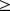 50 Hz), DSI was observable even with relatively small duration stimulations (0.5 sec), whereas, for frequencies less than 50 Hz longer stimulation durations were required in order to evoke an observable DSI. Identifying the exact conditions under which DSI is evoked is crucial in understanding how exogenous CB modulates the activity of the hippocampal network and in which circumstances the network itself recruits the DSI mechanism as a self-regulatory mechanism. Hippocampal pyramidal neurons have been reported to have relatively low firing rates *in vivo* (10-20 Hz) during behaviourally relevant conditions [89], [90]. Thus, it is important to explore, both theoretically and experimentally, the patterns of activation, either normal (like sharp-wave ripples during memory consolidation) or pathological conditions (like epileptic seizures) that would increase the activation in a sufficient frequency level and duration to evoke the mobilisation of eCBs.

Moreover, we investigated with further simulations the role of the pre-synaptic inhibitory cell's activity in the manifestation of the DSI phenomenon, as suggested by experiments from Földy and co-workers [67]. Our results were in agreement with the experiments demonstrating that the firing rate of the pre-synaptic cell has a regulatory role on the capacity of the synapse to exhibit DSI and its magnitude and, under certain conditions, abolishes DSI completely. Importantly, our findings suggest that this CB-mediated plasticity phenomenon, which serves as a homeostatic local mechanism of synaptic efficacy, can be traced to the underlying mechanism of synaptic facilitation due to pre-synaptic firing (by opening more VGCCs) and depression due to post-synaptic firing (by eCB mobilisation and CB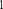 receptor activation).

Plasticity is often manifested as a multi-factor process (see [91] for an extensive review) and there is strong evidence that an important determinant of plasticity is the timing of activity *in vivo*. However, the implications of the relative timing of activity pre- and post-synaptically with regards to DSI modulation have not been investigated experimentally. Having established that DSI is a plasticity phenomenon where both the pre-synaptic firing and the CB availability (often due to post-synaptic firing) are important, we moved on to testing how the relative timing of activity would modulate DSI. Hence, we considered the case of an incoming signal arriving at both cells with delay or advance for a range of frequencies. Our simulations have revealed that, indeed, not only the frequency of the activity of the cell but also its relative timing modulates DSI magnitude. Interestingly, our findings indicate that the causative event for the maximal reduction of DSI is the coincidence of the brief burst of pre-synaptic activity and the peak of CB receptor activation. This model provides testable predictions related to how the activity state of the pre- and the post-synaptic cells, whose bond weight is modulated, determine the occurrence and magnitude of short-term depression of the synapse. The elucidation of such mechanism will have important implications on the effect of CB signalling at the network level. It has already been suggested that CB signalling may serve as a second coincidence detector in the full expression of the canonical STDP curve for short-term plasticity in excitatory terminals [33]. Hence, there could be further consequences when including the CB mechanism in an STDP protocol, for certain brain areas, such as the hippocampus.

### Future Directions

Our findings can be used to guide both future experimental and theoretical studies to confirm our predictions or address unanswered questions. For example, experiments could take place to investigate further the homeostatic modulation of DSI by the pre- and post-synaptic cells' activity in line with our and Földy et al. findings [67]. This could be addressed by varying systematically the pre- and post-synaptic cell firing rates to identify the conditions in which DSI is either recruited or abolished. Our findings also suggest that the variability of DSI, as reported in various studies can be traced to both the dynamics of intracellular calcium (as previously suggested) and also to the sensitivity of the CB receptor to presynaptic firing. Thus, additional experimental studies could also determine the *in vivo* distribution of CB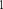 receptor sensitivity to different ranges of pre-synaptic firing, and, investigate whether this sensitivity depends on the prior activity of the pre-synaptic cell. Another key experiment would be to test our predictions on the relative timing of pre- and post-synaptic activity on modulating DSI expression. In fact, the *in silico* protocol (Protocol 8) can be directly tested with experiments to further investigate how the dynamics of the CB receptor activation in concert with the pre-synaptic activation affect the manifestation of DSI. In addition, our model highlights the calcium-driven and PLC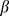-independent pathway in 2-AG synthesis and the lack of a clear understanding how this is manifested. Hence, it is crucial to identify the missing link enzyme/pathway that underlies the calcium contribution to DSI by experiments in order to uncover the characteristics of DSI.

A natural theoretical extension of this work is to use the DSI synaptic model as a building block to extend the model to the network level in order to formulate and investigate hypotheses regarding the function DSI as a modulator of neuronal activity and brain rhythms during memory and learning tasks. Although our model is based on experimental results in the hippocampus [16], [18], [78], this framework could naturally be extended to describe other well-known short-term CB plasticity phenomena, like DSE in hippocampus and DSI/DSE in cerebellum, striatum and spinal cord, where eCBs are also modulating neuronal activity [8].

### Conclusions and Significance of Findings

Since too much or too little CB activity can be harmful, the eCB system is considered to have an important role as a general homeostatic modulator of the molecular plasticity of synapses. Therefore, understanding CB signalling at the sub-cellular level will provide insight on the role of CBs over a range of emergent neural phenomena underlying learning and memory processes, from short- and long-term synaptic plasticity phenomena and spike train modulation to control of coherent brain states and rhythms. Modelling the relatively unique mechanism of CB retrograde signalling is a particularly novel approach in computational neuroscience and an outstanding open challenge. Overall, our model is the first one to formalise and highlight the potential factors controlling the generation and release of eCB at post-synaptic sites and the activation of CB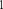 receptors at pre-synaptic sites during DSI. This validated model can be considered as a building block model for studying the effects of eCB in a network study and the physiological role of DSI. Importantly, it provides key insights and predictions on how DSI is modulated by the magnitude and timing of the pre- and post- synaptic cells's activity, which can be further explored both theoretically and experimentally.

## Methods

The description, standard values (unless stated otherwise in the text) and source of parameters are given in Table 1 for the post-synaptic cell and in Table 2 for the pre-synaptic cell. A description of the dynamic variables of the whole model is also provided in Table 3.

**Table 1:** Post-synaptic Cell Parameters.

VARIABLE	DESCRIPTION	VALUE (UNITS)	SOURCE
Ca 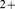 dynamics			
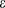	Strength of plasma membrane fluxes	1	Politi et al. [70]
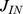	Constant influx	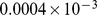 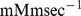	Politi et al. [70]
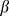	Ratio of effective volumes ER/cytosol	0.185	Politi et al. [70]
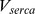	Maximal SERCA pump rate	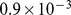 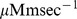	Politi et al. [70]
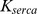	SERCA pump half-activation constant	0.1 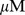	Politi et al. [70]
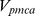	Maximal PMCA pump rate	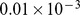 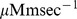	Politi et al. [70]
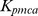	PMCA pump half-activation constant	0.12 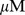	Politi et al. [70]
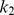	Ca 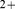 leak rate	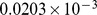 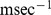	Politi et al. [70]
VGCC			
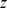	Valence of calcium ion	2	Hemond et al. [9]
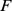	Faraday constant	96485 C/mol	Hemond et al.[9]
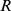	Universal gas constant	8.314 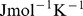	Hemond et al.[9]
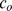	Extracellular calcium concentration	2000 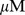	Hemond et al.[9]
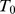	Temperature	25 °C	this model
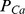	L-type VGCC maximum permeability	0.000275 cm/sec	adapted from [98] and [9]
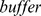	Fast intracellular buffering factor	0.01	[86]
2-AG Synthesis			
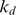	DAG degradation rate	0.66 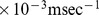	Politi et al. [70]
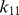	2-AG synthesis rate	0.5 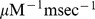	this model
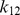	2-AG degradation rate	0.01 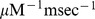	this model
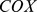	COX-2 availability	1 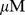	this model
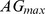	Maximal 2-AG	50 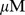	this model
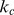	Maximal Ca 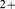 -evoked DAG synthesis rate	10 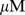	this model
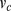	Half-activation constant for Ca 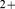 -evoked DAG synthesis	0.7 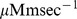	this model
Synapses			
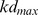	Scaling factor	100 mV	adapted from [7]
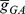	GABA peak conductance	0.3 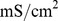	Bertram et al. [7]
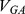	GABA reversal potential	-80 mV	Bertram et al. [7]
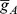	AMPA peak conductance	10 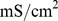	this model
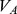	AMPA reversal potential	0 mV	this model
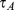	AMPA synaptic time constant	1 msec	this model
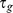	GABA synaptic time constant	1 msec	Bertram et al. [7]

The table describes the parameters for the post-synaptic cell model regarding the dynamics of the intracellular calcium, L-type VGCC, 2-AG synthesis and synapses.

**Table 2:** Pre-synaptic Cell Parameters.

VARIABLE	DESCRIPTION	VALUE (UNITS)	SOURCE
CB 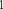 receptor			
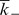	Reluctant-to-willing transition rate	0.3 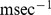	adapted from [73]
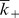	Willing-to-reluctant transition rate	0.0006 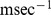	adapted from [73]
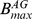	Maximum inhibition (due to AG)	0.5	Guo et al. [76]
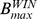	Maximum inhibition (due to WIN)	0.48	Guo et al. [76]
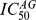	Half-inhibition concentration (AG)	0.48 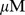	Guo et al. [76]
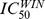	Half-inhibition concentration (WIN)	0.002 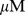	Guo et al. [76]
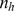	Hill coefficient	1.2	Guo et al. [76]
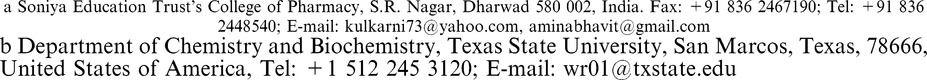	Time constant for unbinding of CB 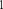 receptor	1000 msec	this model
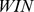	Concentration of WIN55,212 agonist	0 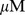	this model

The table describes the parameters for the pre-synaptic cell model regarding the CB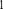 receptor model.

**Table 3:** Dynamic Variables.

VARIABLE	DESCRIPTION	UNITS
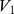	Pre-synaptic membrane potential	mV
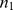	Gating variable	–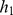	Gating variable	–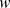	Fraction of willing Ca 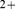 channels	–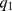	Fraction of bound G proteins (CB 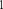 receptors)	–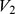	Post-synaptic membrane potential	mV
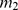	Gating variable	–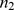	Gating variable	–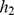	Gating variable	–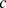	Ca 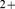 concentration in the cytoplasm	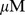
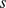	Ca 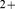 concentration in the ER stores	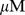
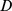	DAG concentration in the cytoplasm	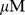
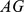	2-AG concentration produced post-synaptically	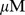
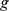	Fraction of bound post-synaptic GABA 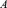 receptors	–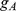	Fraction of bound post-synaptic AMPA receptors	–

The table describes all the dynamic variables of the model.

### Pre-synaptic Inhibitory Cell Model

#### Cell Model

For simulating the pre-synaptic inhibitory cell, we use the single compartment model of Wang-Buzsáki (WB), which describes the activity of hippocampal and neocortical fast-spiking interneurons [92]. The kinetics and maximal conductances, which are of Hodgkin & Huxley [93] style, have been modified in [92] from the original values, so that the model displays two salient features of the hippocampal and neocortical fast-spiking interneurons. The first property is that the action potential in these cells is followed by a brief afterhyperpolarisation. The second property is that the model can fire repetitive spikes at high frequencies. It should be noted that CCK positive interneurons generally have a regular spiking behaviour (with certain exceptions having a fast-spiking profile [94], [95]). Although the WB model is a fast spiking model, this does not pose a problem in the current study. However, for a network study the appropriate model adjustment should take place to limit sustained fast spiking in this neuron model.

The evolution of the membrane potential 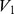 is described by the following current balance equation
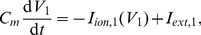
(1)


where 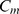 is the membrane capacitance, 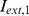 is the external current applied and 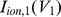 is the total ionic current given by the expression
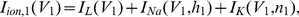
(2)


where
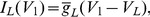
(3)

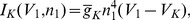
(4)

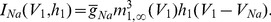
(5)


The gating variables 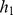, 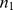 are given by first order differential equations of the form
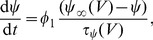
(6)


where 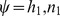 and
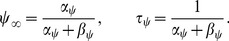
(7)


The gating variable 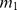 is assumed to be activated instantaneously and is substituted by its steady-state function
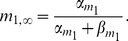
(8)


Further details about 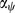 and 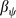 equations (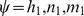) and the parameters of the WB model can be found in [92]. The temperature adjustment variable 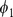 is given by
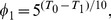
(9)


where 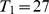 °C denotes the temperature of the original electrophysiological experiment for constructing the WB model. The parameter 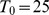 °C denotes the temperature of the model during simulations.

#### CB_1_ Receptor

For the CB_1_ receptor model, we adapt a minimal model for G protein-mediated synaptic facilitation and depression through inhibition of pre-synaptic calcium channels by Bertram and co-workers [73]. The activation of the GPCR (by either hormones or a neurotransmitter) results in the splitting of the G protein heterotrimer into G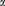 and G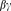 subunits. The G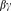 subunit directly inhibits calcium channels, shifting their state from willing to reluctant, whereas strong depolarisation of the cell reverses that shift in channel state [73], [96].

In the original model, the fraction of willing calcium channels, 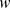, is described by
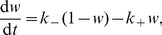
(10)


where 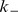 is the reluctant-to-willing transition rate (reflecting the V-dependent dissociation of G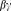 from the channel) and is given by the expression
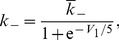
(11)


that is, a sigmoid function which depends on 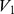 and has a half-maximum value of 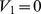 mV.

The 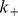 in equation (10) is the willing-to-reluctant transition rate, reflecting the concentration of the activated G proteins (which was constant in the original model). In our adaptation of the Bertram model, the GPCR is considered to be the CB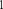 receptor, which is activated by CBs. Hence, 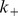 is adapted to be dynamic and dependent on the fraction of bound/activated G proteins (denoted here as 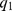) and is given by the expression
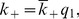
(12)


where 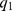 evolves according to
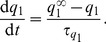
(13)


We point out that since the dynamics of MGL are out of the scope of this study, degradation by MGL is included in the model with a simple coefficient determining the fraction of available 2-AG post-degradation, that is,
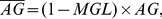
(14)


where 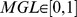 and unless stated otherwise, 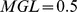. 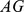 indicates the 2-AG mobilised by the post-synaptic cell (see equation (42)), whereas 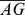 indicates the effective 2-AG, which binds and activates the CB_1_ receptor.

The activation of the CB_1_ receptor by both the endogenously produced CB, namely 2-AG (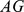), and the exogenously applied agonist, WIN55,212 (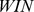), is included in the model. However, the effect of 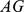 and 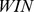 binding on the CB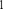 receptor has different concentration response curves for the inhibition of the pre-synaptic calcium channels [76]. Hence, we use the Hill functions fitted to these two concentration response curves (see Figure 2) as 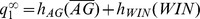 where
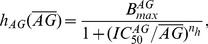
(15)

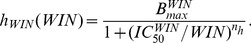
(16)


Here, 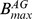, 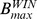 represent the maximum inhibition and 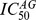, 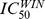 represent the half-inhibition concentration and 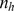 is the hill coefficient. As in [73], we assume no contribution from the calcium channel at the pre-synaptic terminal to the firing of the pre-synaptic cell.

### Post-synaptic Excitatory Cell Model

#### Cell Model

We consider a single compartment model for the post-synaptic pyramidal cell model. The models for the fast Na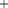 and K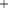 currents responsible for action potentials in hippocampal pyramidal cells are taken from [68] (as modified from [69]) and follow the Hodgkin & Huxley formulation. The evolution of the membrane potential 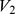 is described by the following current balance equation
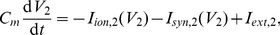
(17)


where 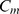 is the membrane capacitance, 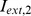 is the external current applied, 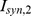 is the total synaptic input current and 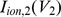 is the total ionic current given by the expression
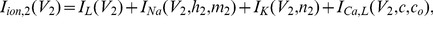
(18)


where
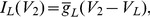
(19)

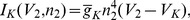
(20)

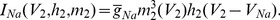
(21)


The gating variables 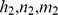 are also given by first order differential equations of the form (6). Further details about 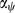 and 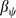 equations (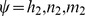) and the parameters of the model can be found in [68] and were obtained from ModelDB (accession number: 3808) [97]. This model is adjusted for temperature with the factor
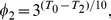
(22)


where 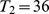 °C denotes the temperature for the original electrophysiological experiment for constructing the model. The parameter 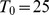 °C denotes the temperature of the model during the simulations.

#### Voltage Gated Calcium Channel

We used the Goldman-Hodgkin-Katz (GHK) flux equation formulation for the Ca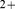 ion [98] to describe the calcium current 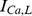 (in equation 18) through the L-type VGCC, that is,
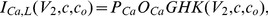
(23)


where 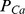 represents the maximum permeability and 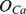 represents the fraction of the open channels and is a function of the gating variables 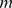 and 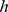, given by
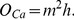
(24)


The driving force equation 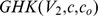 depends on the voltage of the post-synaptic cell, 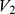, intracellular calcium, 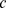, and extracellular calcium, 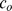, given by
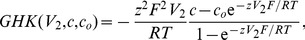
(25)


where 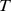 is the absolute temperature, measured in Kelvin ( = T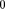 + 273.15), 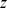 is the calcium ion valence, 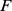 is the Faraday constant and 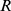 is the universal gas constant (see Table 1). More information on the gating variables can be found in [98].

#### Synapses

The synaptic current for the post-synaptic cell in equation (17) is given by
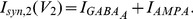
(26)


For the inhibitory plastic GABA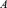 synapse, we use the model from [73],
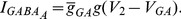
(27)


As in [73] we omit the neurotransmitter release equations and incorporate instead the fraction of willing pre-synaptic Ca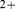 channels (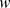) directly into the expression for the fraction of bound postsynaptic GABA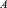 receptors, 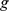, expressed by
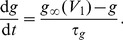
(28)


Hence, g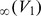 is written as a sigmoid function with a half-maximal voltage (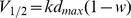) that depends on 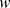, that is,
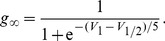
(29)


For example, when all the pre-synaptic calcium channels are in willing state, 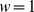, thus, 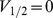 mV and a pre-synaptic action potential results in a large postsynaptic response 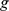. In contrast, when all the pre-synaptic calcium channels are in reluctant state, 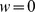, thus, 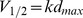 and a pre-synaptic action potential activates a small fraction of postsynaptic receptors resulting in a small post-synaptic response 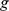. For 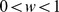 intermediate responses are obtained. Here, 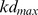 is a scaling factor, given in Table 1. For the excitatory AMPA synapse, we use a standard synaptic model
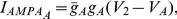
(30)


where 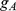 is the fraction of bound post-synaptic AMPA receptors as given by an alpha function.

#### Intracellular Calcium Dynamics

Here, we used an adaptation of an intracellular calcium model by Politi et al. [70]. Assuming that the cell is well mixed (i.e. concentration of calcium is the same throughout the cell), then based on the calcium conservation the concentration of Ca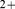 in the cytoplasm and in the ER, denoted by 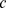 and 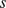, respectively, are described by the following differential equations [70], [99],


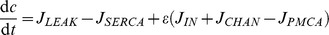


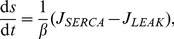
(32)


where the parameter 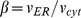 denotes the ratio of the effective volumes of ER volume to the cytoplasmic volume and denotes the strength of plasma membrane fluxes. The 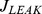 calcium flux represents a leak into the cell (from ER) and is given by
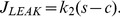
(33)


The 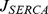 represents the flux to ER through the sarco/endoplasmic reticulum Ca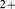 ATP-dependent pumps (SERCA) and is modelled by a Hill's function with a Hill coefficient equal to 2
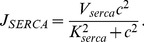
(34)


The 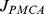 represents the flux to the outside of the cell through the plasma membrane Ca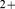 ATP-dependent pumps (PMCA) and is modelled in a similar fashion as the 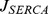,
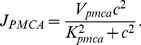
(35)


The constant influx (or leak) of calcium from the outside of the cell is equivalent to 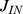 (see Table 1) and the channel flux is 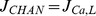 (as expressed in equation (23)). We included an expression for the DAG (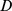) concentration, which is given by,
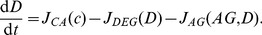
(36)


Here, 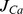 represents the dependency of DAG production on calcium and is modelled by a Hill's function with a Hill coefficient equal to 2,
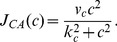
(37)


The 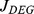 represents the degradation of DAG
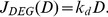
(38)


The 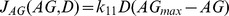 represents the DAG which is metabolised to 2-AG and can be obtained from the kinetic scheme (40).

*Current to Flux conversion.* In order to calculate the current contribution to the increase of calcium concentration, we convert the total calcium current 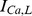 (in 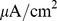) into the chemical flux 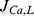 (in 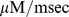) by multiplying the current by a conversion factor 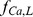 (expressed in m
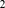
M/mC) such that 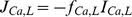. The conversion factor, 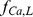, is given by the expression
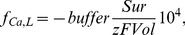
(39)


where 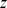 is the calcium ion valence, 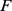 is the Faraday constant and 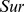 and 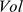 are the surface and volume of the model cell, respectively [86]. For this model (post-synaptic cell), we assume a single compartment with a spherical soma of radius 10 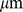. The factor also includes a 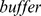 term, which represents the fast intracellular calcium buffering and takes values between 0 and 1, with 1 being equivalent to no buffering and 0 to complete buffering. The remaining factor 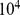 is essential for unit consistency. The minus sign in the expression is a result of defining the membrane currents as positive in the outward direction.

#### eCB Synthesis

We model the synthesis and oxidation of 2-AG in the post-synaptic cell by adding the kinetic reactions 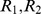,
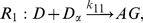
(40)

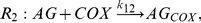
(41)


where 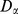 refers to DGL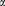, 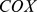 to COX-2 and 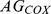 to the product of the oxidation of 2-AG (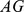) by COX-2. The kinetic schemes, R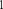 and R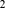, translate to the equation
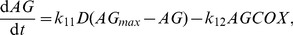
(42)


with the conservation equation 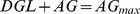.

The release and transport of 2-AG across the synaptic cleft are considered to be fast and are not explicitly modelled. We point out that the dynamics of COX-2 are out of the scope of this study and are not explicitly described. Instead, COX-2 is considered as a constant value acting as a scaling factor and, thus, regulating the production of 2-AG rather than serving as an elimination step for released 2-AG.

#### DSI/eCB-iSTD Measure

The magnitude of depolarisation-induced suppression of inhibitory events was measured as the percentage of the minimum amplitude of IPSPs acquired after the end of the depolarisation relative to the average amplitude of IPSPs acquired before the depolarisation. The depression due to agonist administration was estimated similarly as the percentage of the minimum IPSP amplitude during drug application relative to the average IPSP before drug application. This is expressed as a percentage by
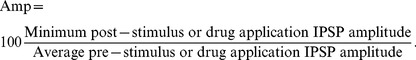
(43)


In the case of drug administration eCB-iSTD = 100-Amp and, respectively, in the case of depolarisation DSI = 100-Amp.

#### Voltage Clamp

In order to model the depolarisation of the membrane potential to a certain value for a certain time interval (as in the experiments), we apply a current 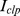 that matches and counteracts the membrane and input currents in order to hold the membrane voltage constant at a certain value 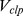, such as
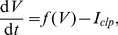
(44)




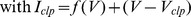
.

### Stimulation Protocols

#### Protocol 1

This experimental protocol is adapted from [78]. Experiments were performed in hippocampal cultures at room temperature. Both the pre- and post-synaptic cells are clamped at –80 mV. The pre-synaptic cell is then depolarised to 80 mV for 2 msec with 0.2 Hz to evoke inhibitory neurotransmitter release. The agonist WIN55,212 of CB_1_ receptors is applied for 1 min (till the steady state of DSI is reached).

#### Protocol 2

This experimental protocol is adapted from [16], [18]. Experiments were performed in hippocampal cultures at room temperature. Both the pre- and post-synaptic cells are clamped at -80 mV. The pre-synaptic cell is then depolarised to 80 mV for 2 msec with 0.2 Hz to evoke inhibitory neurotransmitter release. The post-synaptic cell is depolarised to 0 mV for varying length of time (0.5–5 sec).

#### Protocol 3

This experimental protocol is adapted from [40]. Experiments were performed in hippocampal slices at room temperature. Both the pre- and post-synaptic cells are clamped at –70 mV. The pre-synaptic cell is then depolarised to 70 mV with 0.2 Hz to evoke inhibitory neurotransmitter release (in [40] the stimulating electrode was in or near CA1 stratum pyramidale). The post-synaptic cell is depolarised to 0 mV for varying length of time (0.5–5 sec). The buffering parameter is set to 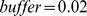. In case 1, DSI is measured with the default method (see “DSI/eCB-iSTD Measure” subsection), whereas, in case 2, DSI is measured as the percentage of the average of 2 IPSPs acquired just after the end of the depolarising pulse relative to the average amplitude of IPSPs acquired before the depolarisation.

#### Protocol 4

Both the pre- and post-synaptic cells are clamped at -80 mV. The pre-synaptic cell is then depolarised to 80 mV for 2 msec with 0.2 Hz to evoke inhibitory neurotransmitter release. The post-synaptic cell is depolarised to 0 mV for varying duration (0.1–10 sec) while either *(i)* the buffering parameter (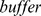) is varied from 0.01–0.1 (equivalent to 99–90%), or *(ii)* the extracellular calcium parameter 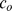 is varied from 500–5000 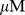).

#### Protocol 5

This experimental protocol is adapted from [18]. Experiments were performed in hippocampal slices at room temperature. The pre-synaptic cell is clamped at -80 mV and is depolarised to 80 mV for 2 msec with 0.2 Hz to evoke inhibitory neurotransmitter release. The post-synaptic cell is stimulated for varying duration and frequency either with *(i)* short (5 msec) depolarising current pulses (the amplitude of the current is adjusted so that an action potential is elicited, 25 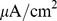), or *(ii)* synaptic excitatory events which stimulate the AMPA synapses (peak synaptic conductance is adjusted so that an action potential can be elicited on the post-synaptic cell).

#### Protocol 6

The pre-synaptic cell is stimulated with short depolarising current pulses (2 msec) with varying frequency (0.2–40 Hz). The amplitude of the current is 25 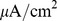. Either the post-synaptic cell is stimulated with a 5 sec depolarising voltage pulse or the agonist WIN55,212 of CB_1_ receptor is applied for 1 min.

#### Protocol 7

The pre-synaptic cell is stimulated with short depolarising current pulses (2 msec) with varying frequency (0.2–50 Hz). The post-synaptic cell is stimulated with short depolarising pulses (5 msec) for varying duration (0.5–5 sec) and 50 Hz frequency. The amplitude of the current in both cases is 25 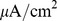.

#### Protocol 8

Both the pre- and post-synaptic cells are stimulated with short depolarising pulses (2 msec for pre-synaptic and 5 msec for post-synaptic cell) for varying duration and frequency. The amplitude of the current in both cases is 25 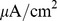. As in experiments with pair recordings, a test current pulse of 0.2 Hz is also applied on the pre-synaptic cell, for assessment of DSI.

### Simulations and Parameters

The system of differential equations was solved numerically using a fourth-order Runge-Kutta algorithm implemented in XPPAUT (http://www.math.pitt.edu/~bard/xpp/xpp.html) [100] and the Python interface for XPP, XXPy, available at http://seis.bris.ac.uk/~enxjn/xppy/. Upon publication, the model will be made available for public download from the ModelDB model repository of the SenseLab database (http://senselab.med.yale.edu/ModelDB/B).
